# Metabolic profiling of adult and pediatric gliomas reveals enriched glucose availability in pediatric gliomas and increased fatty acid oxidation in adult gliomas

**DOI:** 10.1186/s40478-025-01961-w

**Published:** 2025-03-15

**Authors:** Vladislav O. Sviderskiy, Varshini Vasudevaraja, Luiz Gustavo Dubois, James Stafford, Elisa K. Liu, Jonathan Serrano, Richard Possemato, Matija Snuderl

**Affiliations:** 1 Department of Pathology, NYU Langone Health, New York, NY USA; 2 NYU Grossman School of Medicine, New York, NY 10016 USA; 3 Department of Neurological Sciences, Larner College of Medicine, University of Vermont, Burlington, VT USA; 4 Laura and Isaac Perlmutter Cancer Center, New York, NY 10016 USA; 5 Department of Radiation Oncology, Washington University in St. Louis, St. Louis, MO USA; 6 Institute of Biomedical Sciences, Federal University of Rio de Janeiro, Rio de Janeiro, Brazil; 7 Department of Pathology, NYU Langone Health, 240 E 38Th Street, 22Nd Floor, New York, NY 10016 USA; 8 Department of Pathology, NYU Langone Health, 550 First Avenue, Smilow 611, New York, NY 10016 USA

**Keywords:** Metabolic profiling, Pediatric glioma, Adult glioma, H3 mutant, Glucose, Fatty acid oxidation

## Abstract

Gliomas are the most common primary brain tumors and a major source of mortality and morbidity in adults and children. Recent genomic studies have identified multiple molecular subtypes; however metabolic characterization of these tumors has thus far been limited. We performed metabolic profiling of 114 adult and pediatric primary gliomas and integrated metabolomic data with transcriptomics and DNA methylation classes. We identified that pediatric tumors have higher levels of glucose and reduced lactate compared to adult tumors regardless of underlying genetics or grade, suggesting differences in availability of glucose and/or utilization of glucose for downstream pathways. Differences in glucose utilization in pediatric gliomas may be facilitated through overexpression of *SLC2A4*, which encodes the insulin-stimulated glucose transporter GLUT4. Transcriptomic comparison of adult and pediatric tumors suggests that adult tumors may have limited access to glucose and experience more hypoxia, which is supported by enrichment of lactate, 2-hydroxyglutarate (2-HG), even in isocitrate dehydrogenase (IDH) wild-type tumors, and 3-hydroxybutyrate, a ketone body that is produced by oxidation of fatty acids and ketogenic amino acids during periods of glucose scarcity. Our data support adult tumors relying more on fatty acid oxidation, as they have an abundance of acyl carnitines compared to pediatric tumors and have significant enrichment of transcripts needed for oxidative phosphorylation. Our findings suggest striking differences exist in the metabolism of pediatric and adult gliomas, which can provide new insight into metabolic vulnerabilities for therapy.

**Supplementary Information:**

The online version contains supplementary material available at 10.1186/s40478-025-01961-w.

## Introduction

Gliomas are the most common primary brain tumors with a wide range of molecular drivers and clinical outcomes. Glioblastoma IDH wild-type (GBM) is the most common primary malignant brain tumor in adults, has poor outcomes, and is classified as Grade 4 by the WHO [1]. The median age of diagnosis is 65 with incidence increasing with age and reaching a peak at 75–84 years [2]. Pilocytic astrocytoma is the most common pediatric glioma and represents the slowest growing form of astrocytoma and concordantly are classified as Grade 1 [1]. However, malignant gliomas can also originate in pediatric patients, in whom these tumors have substantial morbidity and mortality with a 5-year survival of under 20% [2]. Genetic profiling of pediatric high grade glioma (HGG) identified a subset that harbor a histone H3 mutation that drives malignant transformation with the most common mutations being a substitution of lysine at position 27 to a methionine (K27M) and a substitution of a glycine at position 34 to either arginine or valine (G34R/V) [3, 4]. These substitutions change the epigenetic landscape of these tumors, allowing for a highly proliferative state that leads to a particularly poor prognosis [5]. For example, pediatric K27M glioma tumors have a survival of less than one year, partly because no effective treatment options exist for these tumors [6]. Further characterization of these tumors is necessary to identify novel vulnerabilities that may be exploited for targeted therapies.

Prior molecular characterization of adult and pediatric tumors suggests that genetically and epigenetically these tumors differ considerably [7, 8]. However the metabolic differences between adult and pediatric tumors has not been assessed and may represent an opportunity to better understand these subgroups and identify novel therapeutic targets. Indeed, we have previously shown that high systemic glucose levels have different prognostic impact in patients with different molecular subtypes of GBM [9]. Metabolic rewiring has been identified as a hallmark of cancer due to the increased demand for macromolecular biosynthesis triggered by oncogenic signaling and required for cell proliferation [10]. The alterations in metabolic pathways in cancer cells permit survival under the pressures of the tumor microenvironment, where unreliable vasculature and increased metabolite consumption often result in a nutrient-poor environment. These same adaptations can create targetable liabilities [11].

Separate metabolomic methods enable the detection of small biochemical intermediates (commonly referred to as polar metabolites) and hydrophobic species, predominantly membrane components (lipid profiling). These methods enable measurement of the relative abundance of the detected metabolite. However, the interpretation of an increase or decrease of a particular species requires contextual information about the levels of other metabolites and gene expression changes, as alteration in either consumption or production can produce the same effect on the level of a given metabolite.

Here, we perform metabolic profiling on 114 adult and pediatric brain tumors and integrate this profiling with transcriptomics to identify key metabolic differences between different types of gliomas. We demonstrate that striking metabolic differences exist between adult and pediatric gliomas irrespective of molecular drivers or histological grade. We show that pediatric tumors are enriched for glucose metabolism while adult tumors seem to be more dependent on fatty acid oxidation (FAO).

## Materials and methods

### Adult and pediatric specimen collection and storage

All tumors were newly diagnosed and collected fresh prior to any adjuvant therapy and frozen in − 80^ °^C. Age of 18 years was used to separate tumors into adult and pediatric category, with two tumors that were not grouped accordingly. A case of Pleomorphic Xanthoastrocytoma (PXA) in a young adult was grouped with Pediatric Other and one young adult with a GBM was grouped with pediatric GBM RTK. Individual sample information including subgroup labels are noted in Supplementary Table 1.

Pathology diagnosis was retrieved from the medical record. All tumors were profiled and classified using clinically validated whole genome DNA methylation profiling and v11 version of the DKFZ brain tumor classifier [12] as described previously [13] and DNA methylation class retrieved from medical records to confirm the diagnosis and methylation subtype. Only samples without discrepancies between histopathologic diagnosis and DKFZ classifier were included. For metabolomic and RNA expression analysis, all adult cases classifying as GBM were grouped as Adult GBM. All IDH mut ant cases, irrespective of astrocytic or oligodendroglial origin were grouped together as Adult IDH as the number of cases of each subtype precluded further stratification. Both K27M and G34 mutant gliomas were grouped together as Pediatric High-Grade Glioma- histone mutated (HGG-H3). All pediatric glioblastomas receptor tyrosine kinase (RTK) subgroups were grouped as Pediatric GBM -RTK, and no Mesenchymal subtype tumors were present in this subgroup. Other rare tumors, such as Pleomorphic Xanthoastrocytoma (PXA), Subependymal Giant Cell Astrocytoma (SEGA), Ganglioglioma and Ependymoma were grouped together as Pediatric-Other. For detailed clinical and pathology data see Supplemental Table 1.

### Metabolite profiling and metaboanalyst batch correction

Metabolite profiling was performed as previously described [9]. Briefly, approximately 10–20 mg of each tumor specimen was processed sequentially in LC/MS grade methanol, water, and chloroform before homogenization using a Bertin Precellys 24 homogenizer. The lipid and polar layers were collected separately and SpeedVac dried. Dried metabolite samples were stored at -80 °C prior to analysis. MetaboAnalyst (version 3.2.0) R package was used as previously described for data preprocessing, batch correction, and differential metabolomic analysis of polar and lipid metabolites separately [9, 14]. After batch correction, 128 polar metabolites and 406 lipid metabolites were analyzed in the adult versus pediatric comparison. Normalized batch corrected data was used to perform unsupervised principal component analysis (PCA) and generate heatmaps by examining variance and calculating Z-scores respectively. Normalization was performed across each comparison. Heatmaps were produced using *ComplexHeatmap* R package.

### RNAseq and gene set enrichment analysis

Approximately 10 mg of tumor tissue was allocated for transcriptomic analysis. RNA extraction was performed using the Qiagen RNeasy Lipid Tissue Mini Kit (Catalog #74,804), followed by library preparation and pooling. Sequencing was carried out on the Illumina HiSeq 2500 platform, generating single end reads. The resulting FASTQ files were aligned to the human reference genome (hg19) using the *STAR aligner* [15]. Sample quality was assessed for contamination using *Fastq Screen*, and alignment metrics were generated with *Picard RNASeqMetrics*. Gene expression levels were quantified using *featureCounts* [16].

The raw gene counts were normalized using the variance stabilizing transformation (vst) function within the *DESeq2* R package [17] to enable downstream analyses. Normalized gene expression data were analyzed to investigate a curated list of transcripts encoding metabolic enzymes [18], with heatmaps visualized using the *pheatmap* R package. Differential expression analysis was conducted with DESeq2, and volcano plots were created using the *EnhancedVolcano* R package [19] to identify expression differences between cohort groups. Gene set enrichment analysis (GSEA) was performed using version 4.0.1 of the GSEA software with 1,000 permutations, focusing on curated and hallmark gene sets, as described previously [20–22].

### Statistical analysis

Statistical analysis for metabolites was performed through Metaboanalyst [9, 14] and for transcripts through DESeq2 [17]. Results for further subgroup comparisons were performed using Welch’s t-test in GraphPad Prism software. Significance was defined as a *p*-value < 0.05 for single comparisons or an FDR < 0.05 for multiple comparisons.

## Results

### Metabolic and transcriptomic profiling of adult and pediatric gliomas

Prior work suggests that clear differences exist between the genetic and epigenetic profiles of pediatric and adult gliomas [7, 8, 23, 24]. To elucidate the major differences in metabolic landscape of adult and pediatric gliomas, we performed LC/MS-based profiling of small molecule metabolites and lipid species and integrated it with whole-transcriptome analysis (RNAseq). In total, we profiled 114 tumors, 59 adult tumors and 55 pediatric tumors with subgroups of adult glioblastoma (GBM) IDH wild-type (49 samples), adult astrocytoma isocitrate dehydrogenase (IDH) mutant (8 samples), pediatric high grade glioma (HGG) H3 mutant (18 samples)—K27M (14 pontine samples, no supratentorial samples were available for analysis) or G34V mutant (4 samples), pediatric glioblastoma, which have a signature of overactive receptor tyrosine kinase (RTK) signaling (9 samples), pediatric pilocytic astrocytomas (22 samples), and a mixed group of other pediatric tumors (8 samples), which included ependymomas, subependymal tumors, and gangliogliomas (Sup Table 1).

For analysis using MetaboAnalyst batch correction, we integrated the metabolomics data between the two groups to obtain a normalized metabolite dataset that was used for further analysis. Normalization was performed for each individual analysis. Moreover, we performed RNAseq on a subset of matched tumors with available tissue and analyzed the whole transcriptome, focusing on a curated subset of transcripts encoding metabolic enzymes and small molecule transporters [18]. In total, we profiled 48 tumors, 21 adult GBM tumors and 27 pediatric tumors with subgroups of pediatric high grade glioma (HGG) H3 mutant (11 samples), pediatric glioblastoma—RTK (5 samples), pediatric pilocytic astrocytomas (9 samples), and a mixed group of other pediatric tumors (2 samples, Sup Table 1). Our overarching goal was to integrate the metabolic and transcriptomic data to elucidate the metabolic landscape of adult and pediatric gliomas and identify key metabolic differences between subtypes.

### Pediatric gliomas are more abundant in glucose compared to adult tumors, which have hallmarks of a hypoxic microenvironment

We initially sought to identify groups for comparison with the largest metabolic differences by performing principal component analysis (PCA) for the polar and lipid metabolites separately. Strikingly, PCA of the polar and lipid metabolites demonstrated clear separation between adult and pediatric samples primarily based on the first principal component (PCA1), especially in the lipid metabolite data (Supplementary Fig. 1). There was more divergence in the PCA plots between adult and pediatric tumors than between individual subgroups, suggesting that more significant differences in metabolic drivers exist in the adult versus pediatric comparison than any individual subgroup comparison. For this reason, our initial analysis of our metabolite and transcript data focused on comparing adult and pediatric tumors.

**Fig. 1:**
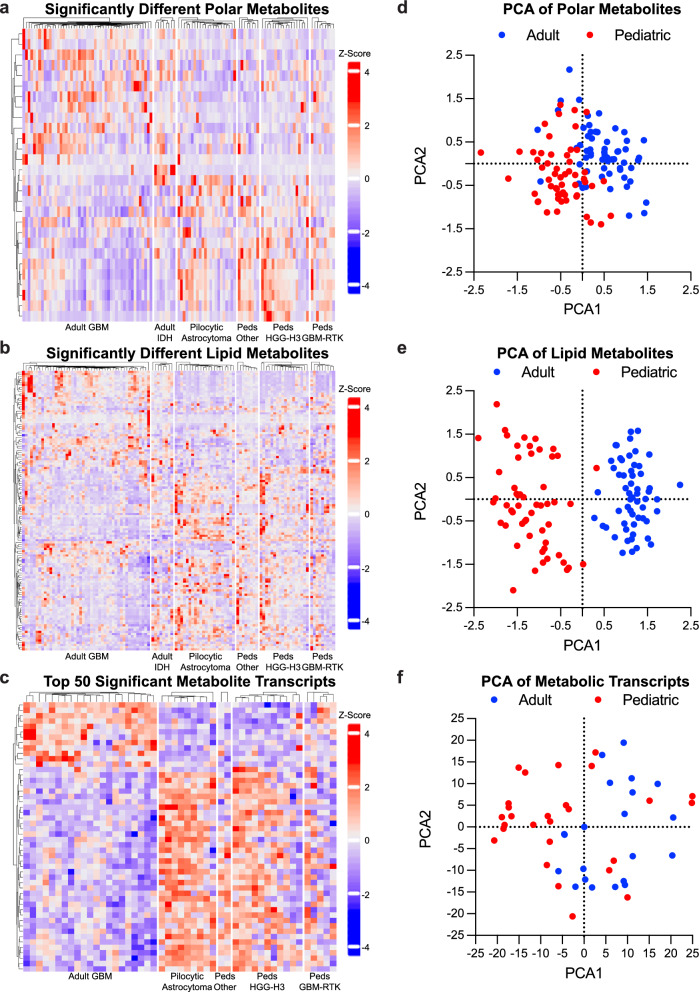
Metabolic Differences Between Adult and Pediatric Gliomas. (a and b) Heatmap reporting the relative abundance of significantly enriched (FDR < 0.05) polar (**a**) and lipid (**b**) metabolites in adult or pediatric gliomas after normalization of batch corrected data. Metabolites presented in order of hierarchal clustering results (left). Samples are grouped by subtype (bottom). The scale bar (right) denotes the Z-score relative to the mean for each metabolite. **c** Heatmap reporting the relative abundance of the top 50 significantly enriched metabolic transcripts in adult or pediatric gliomas (FDR < 0.05). Transcripts presented in order of hierarchal clustering results. Samples are grouped by subtype (bottom). The scale bar (right) denotes the Z-score relative to the mean for each transcript. (**d**-**f**) PCA of normalized batch corrected polar (**d**) and lipid (**e**) metabolite data and transcriptomic (**f**) data. Graphs created using PCA1 and PCA2. Adult samples are depicted in blue and pediatric samples are depicted in red.* Detailed sample and subgroup information can be found in Methods and Supplementary Table 1. Supplementary Fig. 1 has polar (**a**) and lipid (**b**) data subdividing each sample for per the samples subgroup

**Fig. 2:**
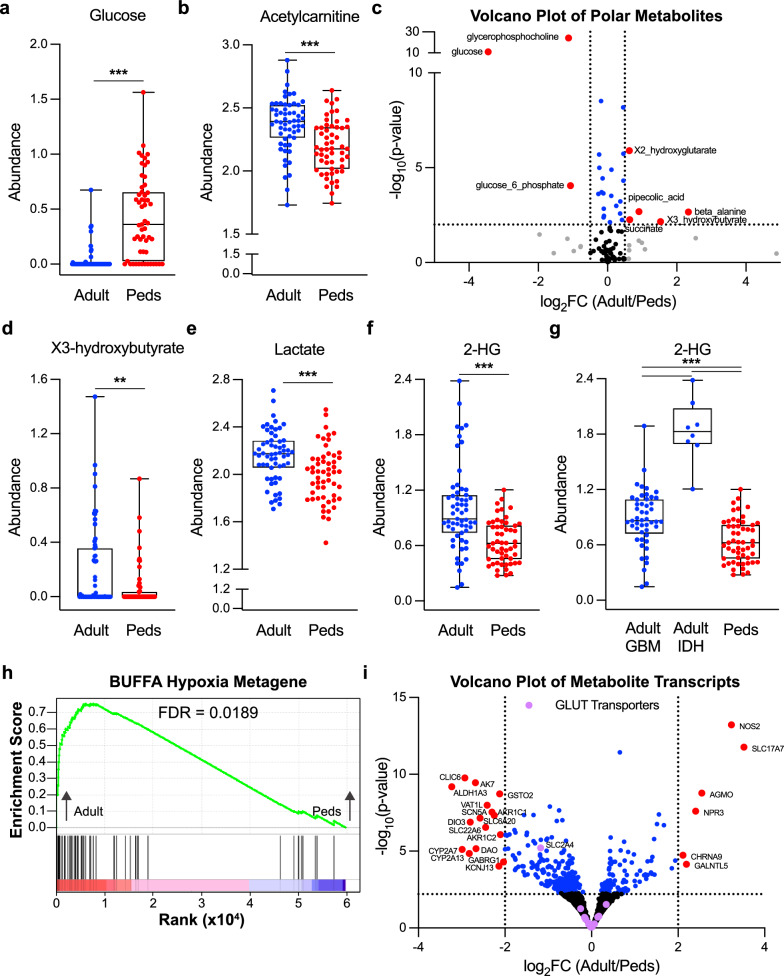
Pediatric Gliomas are Enriched in Glucose Whereas Adult Tumors have Hallmarks of a Hypoxic Microenvironment. **a**, **b**, **d**–**g** Relative abundance of glucose (**a**), acetylcarnitine (**b**), 3-hydroxybutyrate (**d**), lactate (**e**), and 2-hydroxyglutarate (**f** and **g**) in individual adult (blue) and pediatric tumors (Peds, red) using the batch corrected data normalized across all samples. ** denotes *p* < 0.01, *** denotes *p* < 0.001. **c**, **i** Volcano plot depicting significance (-log_10_(p-value) and relative abundance in adult versus pediatric samples (log_2_FC) for each polar metabolite (**c**) or metabolic transcript (**i**). Dashed lines denote cut-offs for significance (horizontal, FDR < 0.05) and enrichment (vertical, log_2_FC > 0.5 or < -0.5 for polar metabolites and > 2 or < -2 for metabolic transcripts). Metabolites or transcripts with significance and enrichment are in red, significance only in blue, enrichment only in gray, and neither in black. Purple in (**i**) denotes GLUT transporters. **h** Output of GSEA analysis for Buffa Hypoxia Metagene. Input is ranked fold change of transcripts in adult relative to pediatric gliomas. The green line denotes enrichment score along the ranked gene set. Black lines denote locations of genes in the gene set within this ranked list. FDR values are indicated

We identified polar metabolites, lipid metabolites, and metabolic transcripts that were significantly different between the two groups and performed hierarchical clustering on these metabolites and transcripts. These analyses identified key metabolic differences between molecularly different types of adult and pediatric gliomas (Fig. 1a–c, Sup Table 2). As before, principal component analysis showed clear separation between adult and pediatric samples in all three datasets, indicating robust underlying metabolic and transcriptional differences (Fig. 1d–f, Sup Table 2). In the polar metabolite analysis, two of the top contributors to PCA1 include glucose and acetylcarnitine, which are elevated in pediatric and adult gliomas respectively (Fig. 2 a–b, Sup Table 2). Glucose has the largest fold change of any significantly different polar metabolite (Fig. 2c). As intratumoral glucose is almost exclusively extracellular, this difference in glucose levels between the two groups of gliomas suggest that either the availability of glucose or its utilization in downstream pathways differs between adult and pediatric tumors. In support of differences in glucose availability, we find that one of the key metabolites elevated in adult tumors is 3-hydroxybutyrate, a ketone body that is produced upon oxidation of fatty acids and ketogenic amino acids under conditions of glucose scarcity (Fig. 2c, d). Moreover, we find that lactate and 2-HG (X2-hydroxyglutarate), metabolites that are elevated as a consequence of mitochondrial inhibition and in hypoxic environments [25, 26], are more significantly enriched in adult tumors compared to pediatric tumors (Fig. 2e, f). Our analysis included adult IDH mutant tumors which have a somatic mutation in isocitrate dehydrogenase that produces the D-enantiomer of 2-HG. 2-HG in IDH mutant tumors acts as an oncometabolite that drives pathogenesis [27], and therefore, we initially hypothesized that the difference we see in adult and pediatric tumors is driven by the adult IDH mutant tumors. However, a comparison upon separation of adult IDH mutant tumors from the rest of the adult samples reveals that adult tumors still have enrichment over pediatric tumors and that adult IDH mutant tumors expectantly have the highest levels of 2-HG of the subgroups (Fig. 2g). We hypothesize that the L-enantiomer of 2-HG, which increases in hypoxic environments [20], predominates in the adult IDH WT tumors compared to pediatric tumors, but are unable to verify the specific enantiomer with our current data due to LC/MS being blind to chirality. Our findings suggest adult tumors experience a more hypoxic tumor microenvironment devoid of glucose. Genes set enrichment analysis (GSEA) of transcriptional targets supports this idea as transcripts associated with hypoxia are enriched in adult gliomas (Fig. 2h), although these differences may be driven by inclusion of more low-grade tumors in the pediatric group. In addition to having a microenvironment more conducive to glucose availability [28], pediatric tumors may also select for increased glucose uptake. Our transcriptional analysis demonstrates that *SLC2A4*, which encodes the insulin-stimulated glucose transporter GLUT4, is among the most highly enriched transcripts in pediatric gliomas compared to adult, (Figs. 1c, 2i) while other GLUT transporters are not significantly different (Fig. 2i). Altogether, these data suggest that pediatric gliomas differ in their metabolism of glucose and that glucose uptake or utilization are key drivers in the metabolite profiles of pediatric versus adult tumors.

### Adult gliomas have increased levels of acylcarnitines and hallmarks of increased oxidative phosphorylation

**Fig. 3:**
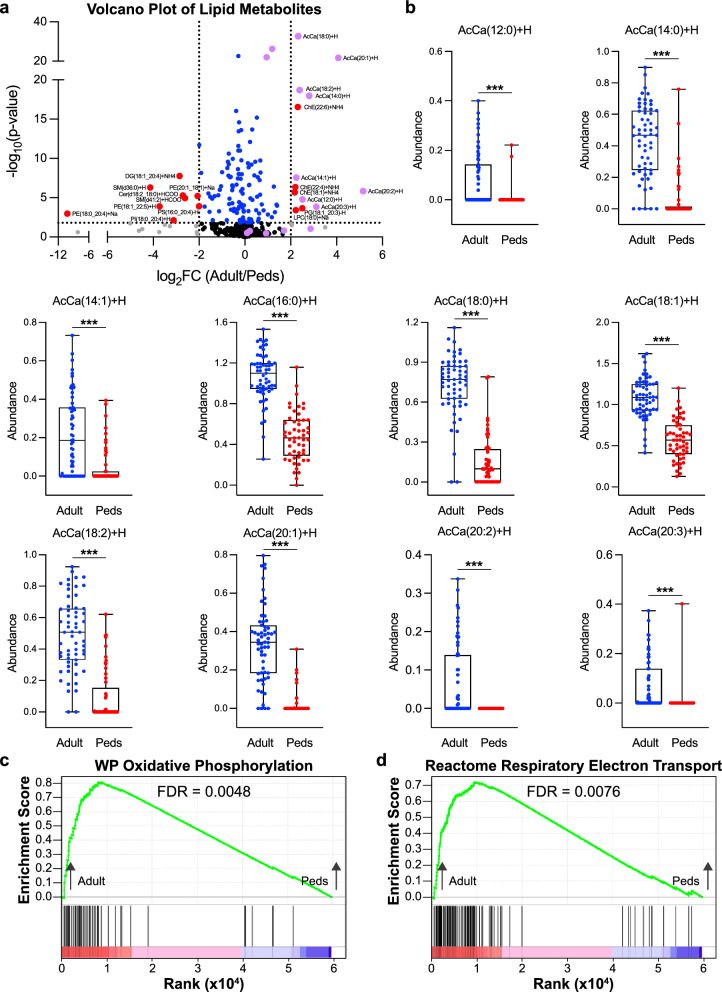
Adult Gliomas have Increased Levels of Acylcarnitines and Hallmarks of Increased OXPHOS. **a** Volcano plot depicting significance (-log_10_(p-value) and relative abundance in adult versus pediatric samples (log_2_FC) for each lipid metabolite. Dashed lines denote cut-offs for significance (horizontal, FDR < 0.05) and enrichment (vertical, log_2_FC > 2 or < -2). Metabolites with significance and enrichment are in red, significance only in blue, enrichment only in gray, and neither in black. Purple denotes acylcarnitines. **b** Relative abundance of indicated acylcarnitines in individual adult (blue) and pediatric (Peds, red) tumors using the batch corrected data normalized across all samples. *** denotes p < 0.001. (c and d) Output of GSEA analysis for WP Oxidative Phosphorylation (**c**) and Reactome Respiratory Electron Transport (**d**) gene sets. Input is ranked fold change of transcripts in adult relative to pediatric tumors. The green line denotes enrichment score along the ranked gene set. Black lines denote locations of genes in the gene set within this ranked list. FDR values are indicated

One of the top polar metabolite contributors to PCA1 distinguishing adult and pediatric glioma is acetylcarnitine, a metabolite that is produced during fatty acid oxidation. Similarly, many of the top contributors to PCA1 of the lipid data are acylcarnitines, all of which are significantly increased in adult gliomas (Fig. 3a, b). Acylcarnitines are intermediates of mitochondrial fatty acid oxidation and the increased levels of these metabolites suggest that adult tumors have increased utilization of fatty acid oxidation for energy production, likely as a result of the glucose deprived microenvironment, which is characteristic for adult GBM. In support of this hypothesis, GSEA demonstrates that oxidative phosphorylation (OXPHOS) and the electron transport chain are among the most highly enriched gene sets in adult tumors compared to pediatric (Fig. 3c, d, Sup Table 3). In pediatric tumors, many of the enriched sets of transcripts are immunologic signatures, likely resulting from the contribution of immune cells to the overall transcriptional signature and suggesting that pediatric tumors may have a less immunosuppressive tumor microenvironment or may otherwise recruit immune cells to support tumorigenesis (Sup Table 3). These data suggest that adult gliomas utilize mitochondrial fatty acid oxidation to a greater extent than pediatric tumors to support their growth and survival.

### Differences in glucose and fatty acid metabolism between adult and pediatric gliomas are independent of tumor grade

**Fig. 4:**
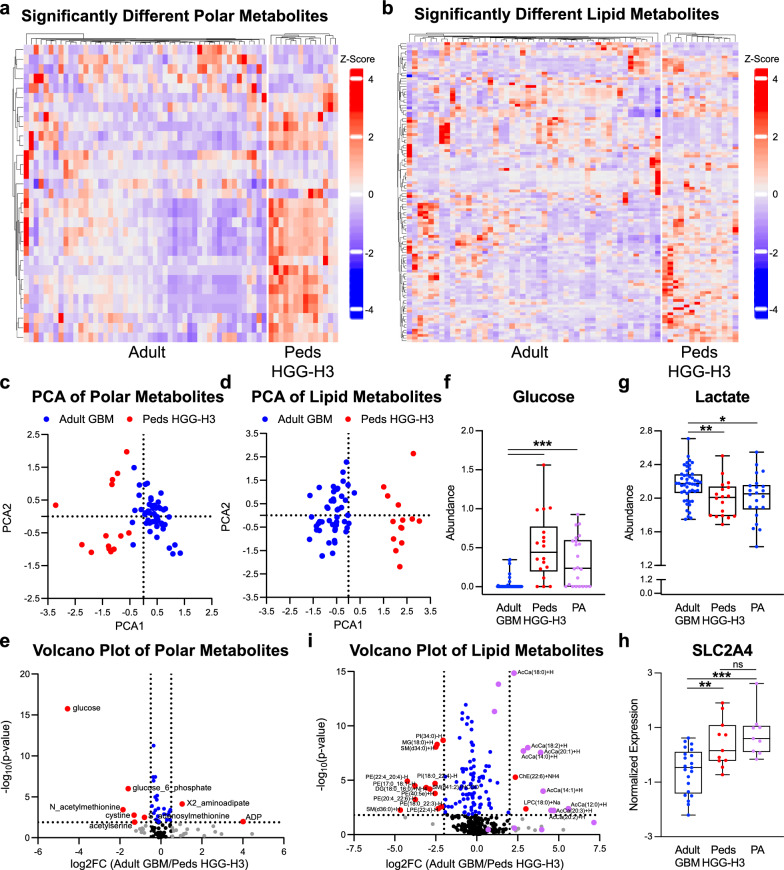
Differences in Glucose and Fatty Acid Metabolism Between Adult and Pediatric Gliomas are Independent of Tumor Grade. **a** and **b** Heatmap reporting the relative abundance of significantly enriched (FDR < 0.05) polar (**a**) and lipid (**b**) metabolites in adult IDH wild-type glioblastoma (Adult GBM) or pediatric high grade H3 K27M or G34V mutant gliomas (Peds HGG-H3) after normalization of batch corrected data. Metabolites presented in order of hierarchal clustering results (left). Samples are grouped by subtype (bottom). The scale bar (right) denotes the Z-score relative to the mean for each metabolite. (c and d) PCA of normalized batch corrected polar (**c**) and lipid (**d**) metabolite data. Graphs created using PCA1 and PCA2. Adult GBM samples are depicted in blue and Peds HGG-H3 samples are depicted in red. **e** and **i** Volcano plot depicting significance (-log_10_(p-value) and relative abundance in Adult GBM versus Peds HGG-H3 samples (log_2_FC) for each polar (**e**) or lipid (**i**) metabolite. Dashed lines denote cut-offs for significance (horizontal, FDR < 0.05) and enrichment (vertical, log_2_FC > 0.5 or < -0.5 for polar metabolites and > 2 or < -2 for lipid metabolites). Metabolites or transcripts with significance and enrichment are in red, significance only in blue, enrichment only in gray, and neither in black. Purple in (**i**) denotes acylcarnitines. **f** and **g** Relative abundance of glucose (**f**) and lactate (**g**) in individual Adult GBM (blue) and Peds HGG-H3 (red) samples using the batch corrected data normalized across all samples. **h** Normalized expression of *SLC2A4* (GLUT4) in Adult GBM (blue), Peds HGG-H3 (red), and pilocytic astrocytomas (PA, purple). * denotes, *p* < 0.05, ** denotes, *p* < 0.01, and *** denotes *p* < 0.001. Analysis of metabolite data was done using batch corrected data normalized across only Adult GBM and Peds HGG-H3 samples except in f and g due to inclusion of PA samples

High-grade pediatric gliomas have poor prognosis similar to adult glioblastomas [2]. Of the high-grade gliomas, H3 mutant tumors have a particularly poor prognosis with a median survival of less than two years [6, 23]. These tumors also share histological features with adult GBM, including microvascular proliferation and necrosis that are associated with hypoxia. Our initial analysis grouped low-grade and high-grade pediatric gliomas together to identify metabolic commonalities of pediatric tumors. However, low- and high-grade pediatric gliomas have markedly different molecular drivers and proliferation rate, and show significant differences in malignant potential and survival. We, therefore, next examined metabolic differences between adult glioblastoma tumors and pediatric HGG H3 mutant tumors to eliminate the influence of lower-grade tumors on our analysis. We again identified metabolites that were significantly different between the two groups and performed hierarchal clustering (Fig. 4a, b). Principal component analysis of both polar and lipid metabolites separated the adult glioblastoma tumors from the pediatric HGG H3 mutant tumors based on PCA1, which had glucose as a key contributor (Fig. 4c, d, Sup Table 4). Indeed, glucose remained the most highly enriched significantly different metabolite (Fig. 4e, f). Adult glioblastoma had diminished glucose levels compared to the pediatric HGG H3 mutants as well as pilocytic astrocytomas. Similarly, lactate remained enriched in adult glioblastoma samples (Fig. 4g); however, 2-HG was no longer enriched, suggesting that 2-HG levels and by extension hypoxia may be influenced by tumor grade. Transcript levels of GLUT4 remain decreased in the adult glioblastoma samples (Fig. 4h). Likewise, acylcarnitines remained significantly abundant in the adult glioblastoma samples (Fig. 4i). Together, these findings suggest that the reliance on fatty acid oxidation compared to glucose is a feature of adult glioblastoma tumors that is not related to its high grade or underlying mutational drivers.

### A comparison of pilocytic astrocytomas to pediatric hgg h3 k27m gliomas demonstrates enrichment in glucose in the h3 gliomas but no difference in acylcarnitines

**Fig. 5:**
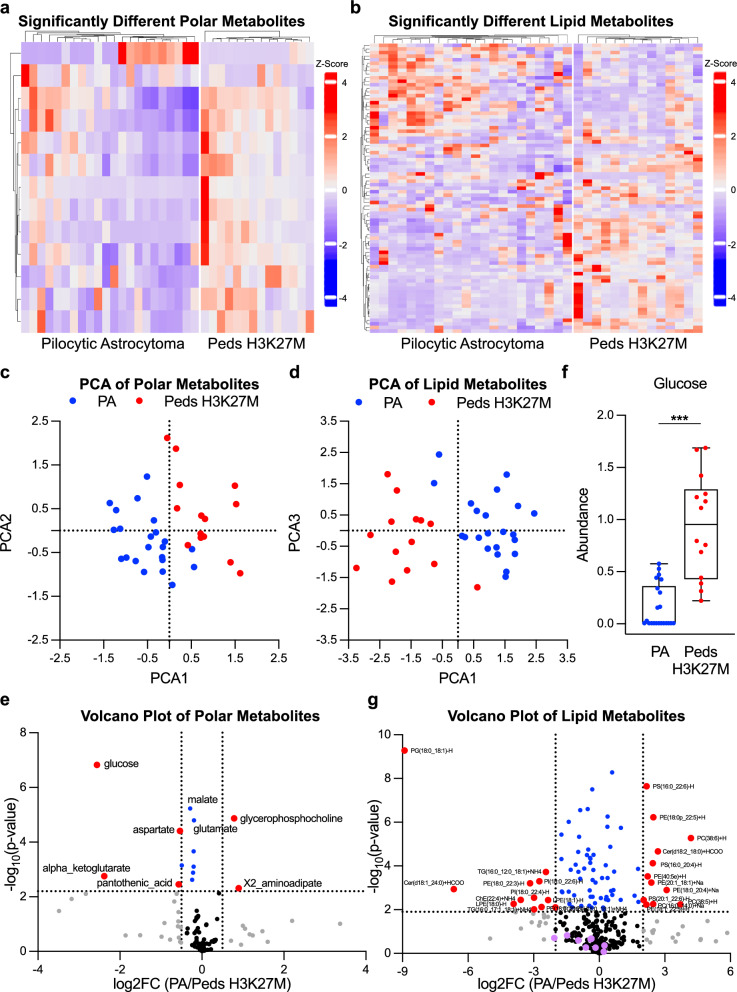
A Comparison of Pilocytic Astrocytomas to Pediatric HGG H3 K27M Gliomas Demonstrates Enrichment in Glucose in the H3 Gliomas but No Difference in Acylcarnites. **a** and **b** Heatmap reporting the relative abundance of significantly enriched (FDR < 0.05) polar (**a**) and lipid (**b**) metabolites in pilocytic astrocytomas (PA) or pediatric high grade glioma H3K27 mutant tumors (Peds H3K27M) after normalization of batch corrected data. Metabolites presented in order of hierarchal clustering results (left). Samples are grouped by subtype (bottom). The scale bar (right) denotes the Z-score relative to the mean for each metabolite. **c** and **d** PCA of normalized batch corrected polar (**c**) and lipid (**d**) metabolite data. Graphs created using PCA1 and PCA2 (**c**) or PCA3 (**d**). PA samples are depicted in blue and Peds H3K27M samples are depicted in red. **e** and **g** Volcano plot depicting significance (-log_10_(p-value) and relative abundance in PA versus Peds H3K27M samples (log_2_FC) for each polar (**e**) or lipid (**g**) metabolite. Dashed lines denote cut-offs for significance (horizontal, FDR < 0.05) and enrichment (vertical, log_2_FC > 0.5 or < -0.5 for polar metabolites and > 2 or < -2 for lipid metabolites). Metabolites or transcripts with significance and enrichment are in red, significance only in blue, enrichment only in gray, and neither in black. Purple in (**g**) denotes acylcarnitines. (**f**) Relative abundance of glucose in individual PA (blue) and Peds H3K27M (red) samples using the normalized batch corrected data. *** denotes *p* < 0.001. Analysis of metabolite data was done using batch corrected data normalized across only PA and Peds H3K27M samples

We next compared pilocytic astrocytoma, the most common low-grade pediatric glioma WHO Grade 1, to the pediatric H3 K27M mutant HGG, WHO Grade 4 to assess metabolic differences between subsets of low- and high-grade pediatric gliomas arising in the same anatomic site, the posterior fossa. As before, we first performed hierarchal clustering of significantly different metabolites and principal component analysis. Our analyses showed that metabolic differences exist between the pilocytic astrocytomas and H3 K27 mutant tumors with separation seen primarily based on PCA1 in both the polar and lipid metabolites (Fig. 5a–d). Interestingly, glucose was enriched in the H3 mutant tumors compared to the pilocytic astrocytomas, which in turn have significantly higher glucose than adult glioblastoma samples (Figs. 5e,f, 4f). We also find that glutamate, alpha-ketoglutarate, malate, and aspartate are enriched in the HGG H3K27M mutant tumors compared to pilocytic astrocytomas (Fig. 5e). These metabolites are linked via the TCA cycle and GOT1/GOT2 transamination reactions, which are required to produce aspartate from glutamine for use in de novo pyrimidine synthesis. The biosynthesis of aspartate is a key function of the mitochondria in proliferating cancer cells, outstripping the importance of its better-known role in ATP generation [29–31]. Elevation of these metabolites might suggest increased glutaminolysis in H3K27M mutant tumors, as glutamine is often the most readily available source of nitrogen in tumors for the synthesis of non-essential amino acids like aspartate. Indeed, inhibitors of glutaminolysis or toxic glutamine analogs have progressed to clinical trials in multiple tumor types. Analysis of lipid data demonstrated that a number of lipid membrane metabolites are differentially enriched between the two subgroups (Fig. 5g). However, acylcarnitines were not differentially abundant, suggesting that activation of fatty acid oxidation is truly a specific feature of adult gliomas, and that there is a hierarchy in the utilization of glucose in adult, high grade pediatric, and low-grade pediatric gliomas.

## Discussion

Despite embodying only 2% of cancers, central nervous system tumors account for the 4th highest life years lost among cancers, are the second most common cancer in children, and the leading cause of cancer-related death in children and young adults [32]. The poor prognosis is largely driven by diffuse gliomas. Most common malignant gliomas such as “glioblastoma IDH wild-type” and “histone mutated high-grade gliomas” have less than an 18-month median survival despite aggressive therapy. Genomic and epigenetic studies have established multiple molecular subtypes of brain tumors [12], including malignant gliomas [24], however therapeutic vulnerabilities remain largely unknown. While pediatric and adult gliomas show distinctly different molecular drivers, tumors of the same grade often share histological similarities, in particular the presence of microvascular proliferation and necrosis with increasing grade. Previous studies have shown that metabolic pathways represent tumor type specific vulnerabilities in glioma. For example, IDH mutant gliomas are highly vulnerable to depletion of the coenzyme NAD +  [33], while Myc activation has been shown to increase dependency on glycolysis in glioblastoma [34].

In this study, we aimed to develop a deeper understanding of the metabolic landscape of adult and pediatric brain tumors. We profiled 114 brain tumors across multiple subtypes and identified that significant metabolic differences exist between adult and pediatric gliomas. We found that the key metabolic feature of pediatric gliomas regardless of grade, was the presence of high levels of intratumoral glucose and reduced lactate. Pediatric gliomas shared these features across all three major groups, HGG H3 mutant, pilocytic astrocytoma and pediatric GBM RTK. Future studies should delineate metabolic differences between K27M mutated, G34 mutated gliomas and other diffuse malignant pediatric high-grade gliomas (RTK and other subtypes) as well as between pilocytic astrocytoma and other low-grade pediatric gliomas. Although the “Pediatric Other” tumor category shared metabolic characteristics with the more abundant pediatric gliomas, a low number of samples and heterogeneity of this cohort, including tumors such as ganglioglioma, PXA, SEGA and ependymoma, precluded further metabolic characterization of these tumors.

The brain depends on glucose metabolism for its physiological function with low levels causing neurocognitive defects. As such, the brain accounts for a disproportional consumption of glucose relative to its size. Uptake of glucose in the brain is primarily mediated by GLUT1 and GLUT3; however, other glucose transporters are expressed in specific regions, including GLUT4, an insulin-sensitive glucose transporter, primarily expressed in muscle and adipose tissue [35]. Within the brain, GLUT4 is expressed in the sensomotor cortex, hypothalamus, cerebellum, hippocampus, and pituitary gland, where its role in these regions remains poorly understood [36]. Recent work suggests GLUT4 plays an important role in glucose tolerance and hypoglycemic counterregulation at the hypothalamus [37] and in memory processing and regulation in the hippocampus [38]. Additionally, GLUT4 mobilization has been demonstrated to play a critical role in supporting energetic demands of neurons at active synapses [39]. Our data demonstrate that pediatric gliomas when compared to adult gliomas have enriched levels of glucose and increased levels of *SLC2A4*, the transcript that encodes GLUT4. Similar to how neurons and muscle tissue utilize GLUT4 to sustain increased metabolic demands, our data suggest that pediatric gliomas, especially the H3 mutant high-grade gliomas, depend on glucose metabolism and may utilize GLUT4 to support the energetic demands of tumor growth, although a dependence on GLUT4 may be tumor microenvironment dependent. Query of the publicly available database DEPMAP demonstrated that GLUT4 is not essential across cell lines or in the sole pediatric glioma cell line in the database, KNS42 [40]. However, this lack of essentiality may be a result of the glucose enriched media in which cell lines are cultured. To fully interrogate the importance of GLUT4 future work will need to explore the transporters dependency in conditions that better represent the tumor microenvironment.

In contrast to the elevated intratumoral glucose levels in pediatric gliomas, adult gliomas were characterized by increased lactate, ketone body 3-hydroxybutyrate, and acyl carnitines, suggestive of FAO dependency. Our observation about a critical role of FAO in GBM is supported by previous studies in cell culture and mouse models [41–43]. For example, inhibition of fatty acid oxidation and SLC22A5, an organic cation/carnitine transporter, downregulation, leads to decreased viability of GBM cells [42]. Moreover, acyl-CoA binding protein (ACBP) has been shown to be highly expressed in adult GBM and promotes GBM proliferation through facilitation of FAO [43]. Our work complements these prior observations and expands its relevance into patient samples. Targeting FAO continues to garner attention as a therapeutic strategy in treating adult glioblastomas that warrants further investigation [44].

Strikingly, compared to pediatric tumors, IDH WT adult gliomas exhibit elevated 2-HG, although this effect may be driven by tumor grade. Multiple dehydrogenases that act on alpha-ketoglutarate can produce 2-HG as an error of metabolism, a phenomenon that can be linked to cancer-intrinsic differences in metabolism as well as environmental effects, such as hypoxia [26, 27]. Both adult and pediatric HGG often display histological features of hypoxia, such as necrosis and microvascular proliferation, yet pediatric HGG H3 mutant tumors showed a higher concentration of glucose and low FAO compared to adult GBM. These data support the notion that intrinsic differences between pediatric and adult gliomas, rather than the environmental response to hypoxia alone, drive these metabolic programs. Indeed, combined transcriptomic and metabolomic analyses support the conclusion that adult glioma has enhanced mitochondrial function. As such, these tumors may be relatively resistant to inhibition of glycolysis. However, adult GBM IDH-wt category is composed of multiple molecular subtypes and future studies should focus on delineating differences between Proneural (RTK1), Classic, (RTK2), Mesenchymal, and other subgroups.

**Fig. 6:**
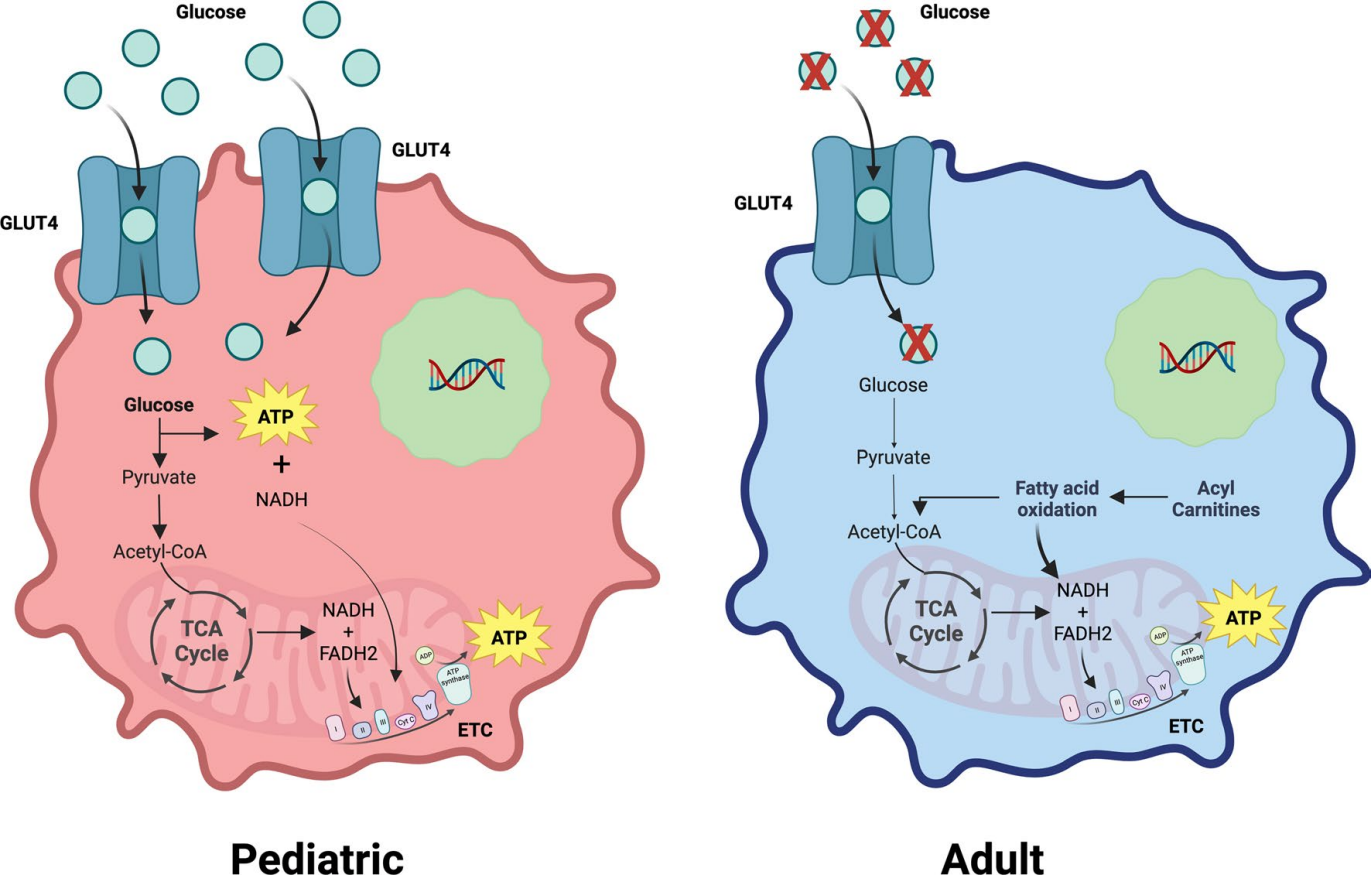
Model Figure. Adult and pediatric gliomas demonstrate key metabolic differences. Pediatric tumors have enriched glucose levels, possibly from increased levels of GLUT4, an insulin-stimulated glucose transporter. Conversely, adult tumors have a hypoxic tumor microenvironment devoid of glucose. Adult tumors instead have increased levels of acyl carnitines, which support ATP production by the electron transport chain through fatty acid oxidation. Our data propose that adult and pediatric gliomas experience unique microenvironments that necessitate separate metabolic drivers to support malignant proliferation

To our knowledge, our study is the first to curate and perform metabolomics on a range of patient derived brain tumor samples spanning pediatric and adult brain tumors. Our data argue that adult and pediatric gliomas exhibit different metabolic landscapes (Fig. 6) and identify multiple key areas for future exploration that may be possible with additional techniques or samples. For example, it would be interesting to delineate the impact of age on the metabolic differences we observe versus whether our findings are more related to tumor intrinsic properties. Similarly, tumor location may be a contributing factor to the metabolic differences that warrants further study. A majority of our pediatric tumors are infratentorial in contrast to the adult tumors, which are primarily supratentorial. Another limitation of our study is the processing of bulk tumors for metabolomic and transcriptomic analysis. Gliomas represent a highly heterogenous tumor population, and future spatial studies would be needed to appreciate the intratumoral metabolic complexity. Future studies will also be needed to make robust metabolic comparisons across the heterogenous population of tumors included in this initial work. Considering these limitations, our data argue that glucose metabolism is particularly important in pediatric gliomas and that the role and importance of GLUT4 warrants further study. Future glucose tracing experiments, to complement our steady-state metabolomics, could uncover downstream metabolic dependencies and vulnerabilities to target in high-grade pediatric gliomas. Conversely, the finding of glucose depletion and elevated OXPHOS in adult glioma tumors begs the question of whether inhibition of fatty acid oxidation could have a meaningful clinical impact. We believe these data and analyses serve as informative tools for future areas of exploration that may impact treatment of brain tumors.

## Supplementary Information

Supplementary Figure 1. Principal Component Analysis Across Sample Subgroups. (a-b) PCA of normalized batch corrected polar (a) and lipid (b) metabolite data. Graphs created using PCA1 and PCA2. Adult IDHm tumors are denoted with an light blue border with oligodendrogliomas depicted with a white core and astrocytomas depicted with a green core, Similarly, Peds HGG-H3 tumors are denoted with a red border with H3 K27M mutated tumors depicted with a white core and G34V tumors with a green core. *Detailed sample and subgroup information can be found in Methods and Supplementary Table 1. Figure 1 contains these same plots with each sample according to whether it was a pediatric (in red) or adult (in blue) tumor. Note that two samples fall into the adult group based strictly on age criteria as shown in Figure but were considered to have pediatric tumors based on pathology. For detailed sample annotation please see Supplemental Table 1.

Supplementary Table 1. Individual Sample Information.

Supplementary Table 2. Supporting Data for Figure 1-3 and 4f, g Focusing on Analysis of Adult Gliomas Compared to Pediatric Gliomas.

Supplementary Table 3. GSEA Results using GSEA Software (version 4.0.1) with 1000 Permutations in the Curated Gene Set and Hallmark Gene Set.

Supplementary Table 4. Supporting Data for Figure 4 Focusing on Analysis of Adult Glioblastomas Compared to Pediatric High-Grade Gliomas H3 Mutant.

Supplementary Table 5. Supporting Data for Figure 5 Focusing on Analysis of Pilocytic Astrocytoma Tumors Compared to Pediatric High-Grade Gliomas H3K27 Mutant.

Supplementary Table 6. Unprocessed Metabolomic and Transcriptomic Data.

## Data Availability

All unprocessed and processed data are available in the Supplemental Tables. RNA seq data are available in GEO (GEO# number: GSE290825).
