# Supplementary figures and images for: Metabolic profiling of adult and pediatric gliomas reveals enriched glucose availability in pediatric gliomas and increased fatty acid oxidation in adult gliomas
Source: Acta Neuropathol Commun. 2025 Mar 15;13:61. doi: 10.1186/s40478-025-01961-w (PMC11909955; doi:10.1186/s40478-025-01961-w)

**a**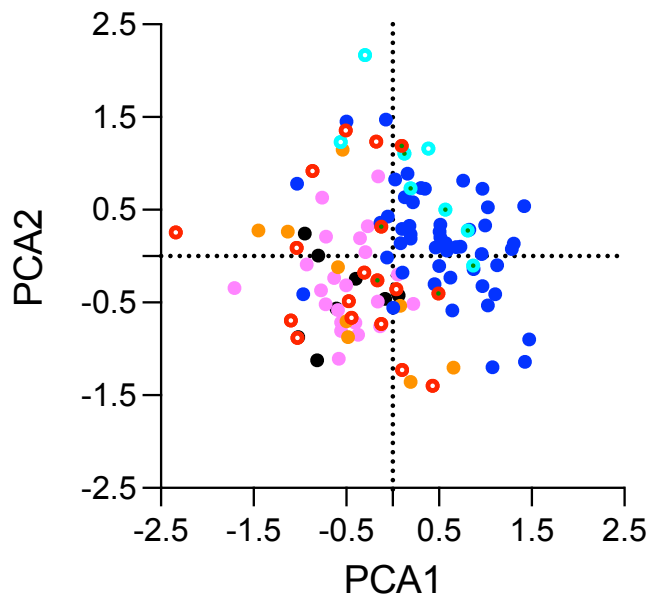**b**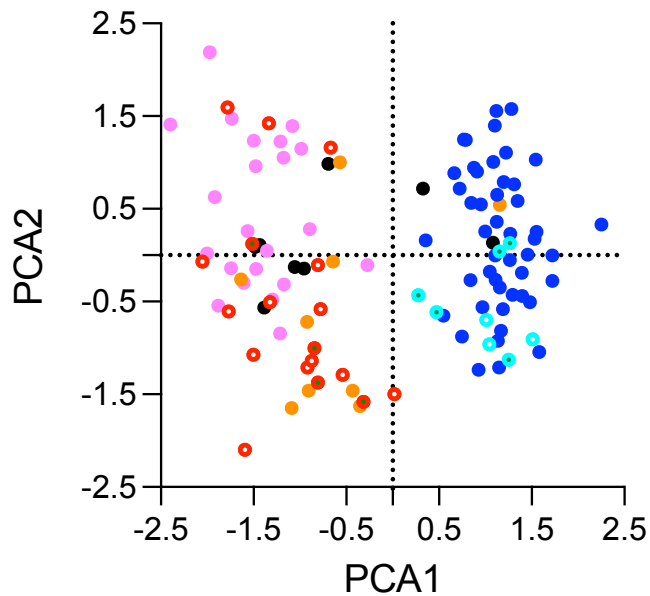

Supplement: Supplementary file 1 — Supplementary Figure 1. Principal Component Analysis Across Sample Subgroups. (a-b) PCA of normalized batch corrected polar (a) and lipid (b) metabolite data. Graphs created using PCA1 and PCA2. Adult IDHm tumors are denoted with an light blue border with oligodendrogliomas depicted with a white core and astrocytomas depicted with a green core, Similarly, Peds HGG-H3 tumors are denoted with a red border with H3 K27M mutated tumors depicted with a white core and G34V tumors with a green core. *Detailed sample and subgroup information can be found in Methods and Supplementary Table 1. Figure 1 contains these same plots with each sample according to whether it was a pediatric (in red) or adult (in blue) tumor. Note that two samples fall into the adult group based strictly on age criteria as shown in Figure but were considered to have pediatric tumors based on pathology. For detailed sample annotation please see Supplemental Table 1. [file 40478_2025_1961_MOESM1_ESM.pdf]
